# Estimating Risk from Ambient Concentrations of Acrolein across the United States

**DOI:** 10.1289/ehp.9467

**Published:** 2006-12-11

**Authors:** Tracey J. Woodruff, Ellen M. Wells, Elizabeth W. Holt, Deborah E. Burgin, Daniel A. Axelrad

**Affiliations:** 1 Office of Policy, Economics and Innovation, U.S. Environmental Protection Agency, San Francisco, California, USA; 2 University of California, San Francisco, San Francisco, California, USA; 3 Department of Environmental Health Sciences, Johns Hopkins Bloomberg School of Public Health, Baltimore, Maryland, USA; 4 Department of Epidemiology and Public Health, School of Medicine, Yale University, New Haven, Connecticut, USA; 5 Office of Environmental Information and; 6 Office of Policy, Economics and Innovation, U.S. Environmental Protection Agency, Washington, DC, USA

**Keywords:** acrolein, dose response, hazardous air pollutants (HAPs), noncancer risk assessment, respiratory function

## Abstract

**Background:**

Estimated ambient concentrations of acrolein, a hazardous air pollutant, are greater than the U.S. Environmental Protection Agency (EPA) reference concentration throughout the United States, making it a concern for human health. However, there is no method for assessing the extent of risk under the U.S. EPA noncancer risk assessment framework.

**Objectives:**

We estimated excess risks from ambient concentrations of acrolein based on dose–response modeling of a study in rats with a relationship between acrolein and residual volume/total lung capacity ratio (RV/TLC) and specific compliance (sC_L_), markers for altered lung function.

**Methods:**

Based on existing literature, we defined values above the 90th percentile for controls as “adverse.” We estimated the increase over baseline response that would occur in the human population from estimated ambient concentrations of acrolein, taken from the U.S. EPA’s *National-Scale Air Toxics Assessment* for 1999, after standard animal-to-human conversions and extrapolating to doses below the experimental data.

**Results:**

The estimated median additional number of adverse sC_L_ outcomes across the United States was approximately 2.5 cases per 1,000 people. The estimated range of additional outcomes from the 5th to the 95th percentile of acrolein concentration levels across census tracts was 0.28–14 cases per 1,000. For RV/TLC, the median additional outcome was 0.002 per 1,000, and the additional outcome at the 95th percentile was 0.13 per 1,000.

**Conclusions:**

Although there are uncertainties in estimating human risks from animal data, this analysis demonstrates a method for estimating health risks for noncancer effects and suggests that acrolein could be associated with decreased respiratory function in the United States.

Previous analyses have shown that estimated ambient concentrations of acrolein, a Clean Air Act hazardous air pollutant, are above levels of concern for noncancer health effects (Caldwell et al. 1998; Tam and Neumann 2004; Woodruff et al. 1998). Estimated ambient concentrations of acrolein exceed the reference concentration (RfC) in > 90% of the 60,000 continental U.S. census tracts for 1990 and 1996; the RfC was exceeded in > 90% of urban census tracts in 1999 [U.S. Environmental Protection Agency (EPA) 2006; Woodruff et al. 1998]. Estimated acrolein concentrations in 1996 and 1999 exceeded 10 times the RfC in > 10% of U.S. census tracts (U.S. EPA 2006). Absent any further regulatory controls, the average ambient concentration of acrolein in the United States in 2030 is projected to be more than three times the RfC (Cook et al. 2006). The RfC is defined as an estimate of continuous inhalation exposure to the human population, including sensitive subgroups, which is likely to be without appreciable risk of deleterious effects over a lifetime (U.S. EPA 2005b).

In humans, acute acrolein exposure produces eye, nose, and throat irritation (Esterbauer et al. 1991; Sim and Pattle 1957; Weber-Tschopp et al. 1977). Acrolein exposure is also recognized to exacerbate asthma (Leikauf 2002); toxicology studies have shown that acrolein exposure results in increased apoptosis of alveolar macrophages (Li et al. 1997), inhibition of neutrophil apoptosis (Finkelstein et al. 2001), increased mucus secretion (Borchers et al. 1999), increased pulmonary edema (Hales et al. 1989; Kutzman et al. 1985), and increased bronchial responsiveness (Ben-Jebria et al. 1994; Leikauf et al. 1989). The current chronic inhalation RfC for acrolein is 2 × 10^−5^ mg/m^3^, based on nasal lesions in rats (U.S. EPA 2003a). Roughly three-fourths of ambient acrolein is estimated to originate from mobile sources (U.S. EPA 2002a), with the remainder from agriculture, industrial processes, tobacco smoke, and forest fires (U.S. EPA 2003b).

Although the RfC for acrolein is based on respiratory system impairment, conventional risk assessment methods applied to animal data do not provide estimates of potential incidence or severity of adverse effects. For example, no particular level of risk is associated with the RfC itself, and interpretation of the significance of concentrations either above or below the RfC is not clear. Except for some environmental contaminants with extensive epidemiologic data, current risk assessment methods for noncancer effects are not amenable to quantifying estimates of potential health effects.

To further elucidate the potential respiratory risks associated with airborne acrolein, we used benchmark dose modeling (BMD) to estimate the probability of respiratory effects at ambient concentrations of acrolein.

## Methods

### Data selection

We reviewed the toxicologic literature to identify data amenable to dose–response modeling, using criteria outlined by the U.S. EPA in its draft *Benchmark Dose Technical Guidance Document* (U.S. EPA 2000). These criteria include the presence of a graded monotonic response with dose and a significant dose-related trend in the selected end point(s) (U.S. EPA 2000). Some of the identified studies did not have sufficient incidence data for modeling (Leach et al. 1987; Lyon et al. 1970). Of the other available studies (Cassee et al. 1996; Costa et al. 1986; Feron et al. 1978; Kutzman et al. 1984, 1985), we selected a data set from Costa et al. (1986), which had a statistically significant dose–response relationship between acrolein exposure and both specific compliance (sC_L_), calculated as dynamic compliance divided by forced residual capacity, and the ratio of residual volume to total lung capacity (RV/TLC). Both sC_L_ and RV/TLC are normalized to an individual animal’s lung size, which corrects for inter-individual variability due to size for these measures.

Full details of the study by Costa et al. are reported elsewhere (1986). Briefly, male Fischer-344 rats were exposed to 0.0, 0.4, 1.4, and 4.0 ppm concentrations of acrolein vapors for 62 days (6 hr/day, 5 days/week). Following exposure, the researchers conducted lung function analyses on 24, 23, 21, and 9 rats from the low to high exposure groups, respectively. The number in the highest dose group was reduced because of high mortality rates (65%) during the experiment; there was no mortality in the other dose groups. There was an additional animal in the 1.4-ppm dose group; however, data on outcomes of interest were not available for this animal, and so it was not included in the present analysis. Residual volume, total lung capacity, and forced residual capacity all significantly increased with increasing acrolein exposure; sC_L_ and RV/TLC also changed significantly with increasing acrolein exposure (Costa et al. 1986). Data from the study were obtained from the authors, and are summarized in Table 1.

### Model development

We converted acrolein concentrations used by Costa et al. (1986) into human equivalent concentrations (HEC) by converting from intermittent exposure to continuous exposure, from shorter to longer duration of exposure, and from rat physiology to human physiology (Supplemental Material available online at http://www.ehponline.org/docs/2006/9467/suppl.pdf). Conversions were based on standard U.S. EPA practice and estimates the U.S. EPA has used previously for acrolein (U.S. EPA 2003b). These HEC values were modeled separately with the two lung function parameters from Costa et al. (1986) using the U.S. EPA’s Benchmark Dose Software (BMDS), version 1.3.2, in accordance with the U.S. EPA’s draft BMD technical guidance document (U.S. EPA 2000). We fit three types of models supported by the BMDS (linear, polynomial, and power) to determine the best-fitting model type. Several criteria were used to determine the best-fitting model, including graphical displays of predicted responses, likelihood ratio tests for model fit, Akaike’s information criterion values, and chi-square residual values at lower experimental doses (Supplemental Material available online at http://www.ehponline.org/docs/2006/9467/suppl.pdf).

### Defining change in continuous end points

**Figure 1:**
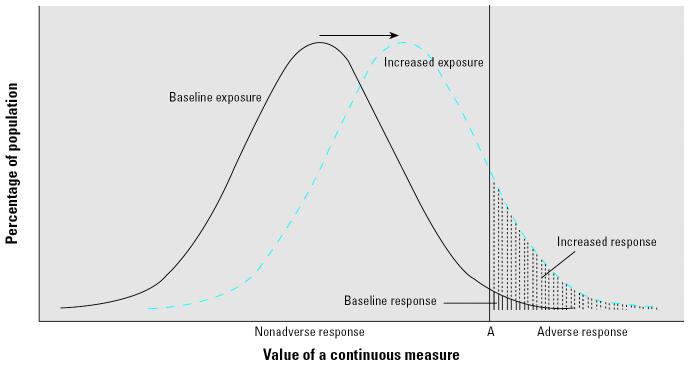
Example of the response distribution among a baseline or unexposed population (solid line) and an exposed population (dashed line). The arrow indicates the change in mean response between the baseline and the exposed populations. Shaded areas represent the proportion of the population with a response past a level “A” that is considered abnormal.

Among a group of individuals, measurements of continuous end points such as ratios of lung function parameters or blood pressure produce a range of values due to inter-individual variation; collectively, these variations result in a distribution of response for the population. Exposure to a toxicant that causes a change in this response will essentially shift the mean and potentially the variance of the resulting response distribution, as shown in Figure 1.

To estimate additional adverse outcomes, or the number of adverse outcomes attributable to acrolein exposure, we need to define what magnitude of a lung function parameter to consider “adverse” (Crump 1995; Gaylor and Slikker 1990; U.S. EPA 2000). After defining the adverse outcome level cutoff (such as level “A” in Figure 1), we can estimate how many individuals in the baseline population are expected to respond at or above this value, and for a population with an increased exposure to a contaminant, the increase in the number of individuals with adverse outcomes over baseline (Figure 1).

There is currently no defined standard for adverse or abnormal levels in sC_L_ and RV/TLC, either in rats or humans. Therefore, we chose two different approaches to estimate adverse health effects. For the first, we defined the baseline adverse outcome prevalence as values ≥ 90th percentile of the control response distribution. Therefore, 10% of unexposed individuals would, by definition, experience adverse outcomes. We refer to this baseline adverse response level as the “adverse cutoff 10” (AC_10_). To estimate the potential effect of acrolein exposure, we estimated the number of additional adverse outcomes expected at a given exposure level. We chose the 90th percentile as our definition of adverse based on consideration of other public health end points. For example, small for gestational age babies are most often defined as those whose birth weight is < 10th percentile of babies with the same gestational age (Tambyraja and Ratnam 1982). For the second approach, we estimated the relative change in lung function parameters following acrolein exposure by determining the relative excess response over baseline mean.

### Estimating additional adverse outcomes

We used methods based on previous work to calculate additional adverse outcomes from continuous, noncancer outcomes (Gaylor and Slikker 1990; Kodell et al. 1995; U.S. EPA 2000). Visually, this value is the difference in the areas between the response distribution curves for exposed and nonexposed populations (Figure 1). This area is determined by converting the response distributions into a standard normal scale and using standard normal deviate (*z*) values to identify their associated probability, or area under the curve.

We defined *p* as the proportion of non-adversely affected individuals in the control population (here, defined at 0.90) and *r* as the proportion of individuals with an adverse outcome in the exposed population. The term *r* can be determined using the equation *k = z_p_*−*z_(p−r)_*, where *z_p_* is the standard normal deviate of the cumulative proportion of non-adversely affected individuals among controls, *z_(p−r)_* is the standard normal deviate for the cumulative proportion of nonadversely affected individuals among the exposed population, and *k* is the multiplier of the standard normal deviate of the increase in mean response, which can be determined from the model for each exposure concentration of interest.

A sample adverse outcome calculation is available in the Supplemental Material (http://www.ehponline.org/docs/2006/9467/suppl.pdf). Calculating within and below the range of the experimental data, we assumed a linear relationship between exposure and response. This is similar to what is assumed in cancer risk extrapolation below the experimental data (U.S. EPA 2005a).

We performed a sensitivity analysis of these results by recalculating the prevalence of adverse outcomes among an unexposed population as being either 2% (AC_2_) or 18% (AC_18_) of the population. Briefly, this corresponds to responses at or above the 98th percentile and 82nd percentile, respectively, of the response distribution. The 98th percentile was chosen because this is typically used by the U.S. EPA for continuous response data (U.S. EPA 2000), and the 82nd percentile was chosen because it is the equivalent distance from the 90th percentile, but in the opposite direction. For sensitivity analyses, we repeated the calculations using *p* = 0.98, *z_p_* = 2.05 for the AC_2_ and *p* = 0.82, *z_p_* = 0.915 for the AC_18_.

We performed a second sensitivity analysis to examine the effect on the results by eliminating the highest dose group in the dose–response modeling. About 60% of the animals died in this group, indicating that the lung function response could be affected by an overwhelming toxicologic response.

### Estimating relative excess response

In the second analysis, the relative excess response for lung function parameter compared to baseline was calculated from the fitted linear dose–response function. The adjusted estimated ambient acrolein exposure levels were used in the selected models (Supplemental Material, Table S2, available online at http://www.ehponline.org/docs/2006/9467/suppl.pdf) to predict the lung function parameters expected following acrolein exposure, or the predicted response. The relative excess response was found by subtracting the baseline response from the predicted response and then dividing the result by the baseline response. The baseline response is defined as the predicted mean lung function parameter ratio at 0.0 ppm acrolein, or 0.08 cm^3^/cm H_2_O for sC_L_ and 0.136 (no units) for RV/TLC.

### Ambient concentrations

We used publicly available data of estimated ambient acrolein concentrations from the U.S. EPA’s 1999 *National-Scale Air Toxics Assessment* (U.S. EPA 2006) to estimate responses to acrolein at ambient concentrations found within the United States. Methodologic details regarding these ambient concentration estimates are further described in the *National-Scale Air Toxics Assessment* (U.S. EPA 2006). Briefly, concentrations were estimated by developing a national inventory of emissions for 1999, which was then used as input to an atmospheric dispersion model (Rosenbaum et al. 1999) that estimated average concentrations for 1999 for each census tract in the United States. Census tracts vary in physical size, but generally contain populations of 4,000–5,000 individuals each.

We used the 5th, 10th, 25th, 50th, 75th, 90th, and 95th percentiles of estimated acrolein concentrations for all, urban, and rural census tracts in the United States to estimate the variation in risks due to acrolein across the country.

The distribution of 1999 annual ambient acrolein concentrations is presented in Table 2. Annual ambient acrolein concentrations exceeded the current RfC for acrolein in > 75%, 90%, and 50% of all U.S. census tracts, census tracts within urban counties, and census tracts within rural counties, respectively.

## Results

**Figure 2:**
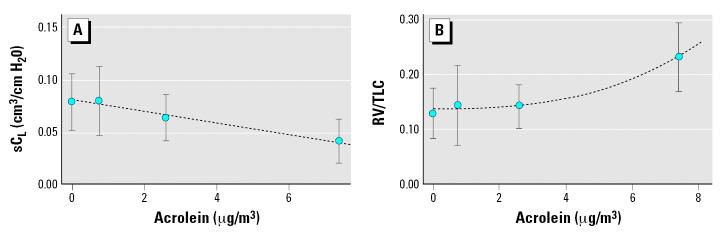
Summary of data from Costa et al. (1986) and model fit for sC_L_ (cm^3^/cm H_2_O) (*A*) and RV/TLC (*B*) for rats in HEC. Error bars indicate 1 SD. The dashed lines represent regression lines from our fitted models: *y*(*x*) = 0.08–0.005*x* for sC_L_; *y*(*x*) = 0.136 + 0.0005*x*^2.63^ for RV/TLC.

Using criteria described elsewhere (U.S. EPA 2000), we selected a linear model (for sC_L_) and a power model (for RV/TLC) as the best-fitting models. Figure 2 displays a plot of model fit and the summary data from Costa et al. (1986). Details of the modeling results are discussed in the Supplemental Material (Table S1; available online at http://www.ehponline.org/docs/2006/9467/suppl.pdf).

**Figure 3:**
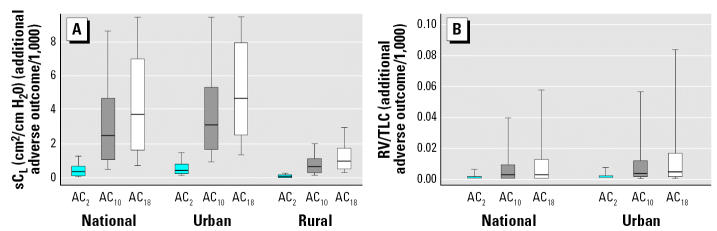
Distribution of additional estimated adverse response (per 1,000) to ambient acrolein using the three different values for defining adverse response. (*A*) sC_L_ (cm^3^/cm H_2_O). (*B*) RV/TLC. Boxes represent interquartile range, horizontal lines represent median, and whiskers extend to the 10th and 90th percentiles. Values for RV/TLC for rural areas are essentially zero and are not shown.

Figure 3 shows the distribution of additional adverse outcomes following exposure to acrolein for the entire U.S. population and for those living in urban or rural census tracts. Estimated ambient acrolein levels are significantly higher in urban areas than in rural areas (*p* < 0.04). A larger change was observed for sC_L_ compared to RV/TLC. We estimated that across the United States in 1999, the median additional adverse outcome was approximately 2.5 per 1,000 for sC_L_ and 0.002 per 1,000 for RV/TLC. Among urban counties, the median estimated excess number of adverse outcomes is 3.1 per 1,000 for sC_L_ and 0.003 per 1,000 for RV/TLC. Among rural counties, these numbers are 0.66 per 1,000 for sC_L_. Additionally, the range of additional adverse outcomes in areas between the 5th and the 95th percentiles of acrolein distribution across census tracts would be 0.28–14 per 1,000 for sC_L_ and 0–0.13 per 1,000 for RV/TLC in all counties, 0.57–15 per 1,000 for sC_L_ and 0–0.16 per 1,000 for RV/TLC in urban counties, and 0.11–3 per 1,000 for sC_L_ and essentially zero for RV/TLC in rural counties. For sC_L_, about 25% percent of U.S. census tracts have an additional adverse outcome prevalence of ≥ 4.6 per 1,000, and about 10% have an estimate of risk of ≥ 8.6 per 1,000.

The estimated additional adverse outcome prevalence per 1,000 people varied when the AC_2_ or AC_18_ were used. For sC_L_, the median amount of additional adverse outcomes using the AC_2_ and AC_18_, respectively, were 0.38 and 3.7 per 1,000 for all counties, 0.48 and 4.6 per 1,000 for urban counties, and 0.10 and 0.99 per 1,000 for rural counties. We found no change in RV/TLC for any county type when the AC_2_ was used, and only minimal changes were observed among urban and all counties when using AC_18_. The full range of additional outcomes resulting from the use of these definitions for adverse effect for the distribution of acrolein exposures across the United States are presented in the Supplemental Material (Table S2; available online at http://www.ehponline.org/docs/2006/9467/suppl.pdf).

We also evaluated the effect on the dose response of eliminating the highest dose group, because there was substantial mortality in this group. Without the high dose group, there was inadequate fit of the RV/TLC response for modeling. The dose–response model for sC_L_ was similar to that with the high dose group, though the slope was slightly steeper. Using the AC_10_ definition for adverse outcome with this model, we found a median additional adverse outcome of 4.2 per 1,000 people among all counties, 5.2 per 1,000 among urban counties, and 1.1 per 1,000 among rural counties. Also according to this alternative model, the range of median additional adverse outcomes between counties at the 5th and 95th percentiles of ambient acrolein exposure in 1999 was 0.48–24 per 1,000 individuals.

The estimated relative excess response in lung function parameters from baseline for the various ambient acrolein concentrations are presented in Table 3. Using the linear model derived from the BMDS, we estimated a 0.53% decrease in sC_L_ associated with median acrolein concentrations, compared with estimates for those without exposure to acrolein. This decrease was 0.65% at the median concentration in urban counties and 0.14% at the median in rural counties. The estimated percent decrease in sC_L_ for those in the 5th to the 95th percentiles of acrolein concentration distribution was 0.06–2.8% for all counties. For RV/TLC, we estimated a 0.0004% increase associated with median acrolein concentrations; the increase at the 95th percentile of acrolein concentration was 0.034%.

## Discussion

This exploratory analysis goes beyond comparisons of ambient concentrations to a reference concentration, instead providing estimates of the number of individuals who may be adversely affected. Acrolein is of particular interest because of indications that much of the U.S. population is routinely exposed to concentrations of acrolein greater than the RfC for this pollutant. We found an estimated median of 2.5 and 0.002 excess cases per 1,000 for additional adverse outcomes in sC_L_ and RV/TLC, respectively.

Lung function impairments are closely related to the manifestation of chronic respiratory disease. Both RV and TLC measurements increase with increasing acrolein exposure, indicating increasing lung volumes. An increase in the RV/TLC ratio suggests that a reduced volume of air is expired. These are consistent with lung function changes seen in obstructive lung diseases, such as asthma, chronic bronchitis, and emphysema (Hlastala and Berger 1996).

Increased lung volume measures are also consistent with decreased compliance (Hlastala and Berger 1996). In their original study, Costa et al. (1986) noted an increase in lung collagen. Increased collagen deposition is part of the airway remodeling process found in chronic asthma; this leads to the narrowing of the airways and potentially reduced airway compliance (Bai and Knight 2005; Haahtela 2001). It is plausible that acrolein exposure could affect collagen deposition; recent work has demonstrated that exposure to other highly reactive airborne toxicants, specifically ozone, alters the expression of fibroblast growth factors (Evans et al. 2003). Reduced airway compliance—in our analysis, measured as sC_L_—may be a compensatory mechanism in response to airway narrowing (Bai and Knight 2005).

Because there were very limited human data available, we modeled lung function responses on toxicologic data in rats. It is possible that acrolein will act in a different physiologic manner in humans and rats, because rats (unlike humans) are obligate nasal breathers. Thus, we do not interpret the lung function measures from the rat study (sC_L_ and RV/TLC) to strictly correspond to specific effects in human lungs. Rather, we take these measured specific effects as more general indicators of potential for decrements in human lung function. Adversity encompasses a range of functional impairment; the U.S. EPA Integrated Risk Information System (U.S. EPA 2005b) defines an adverse effect as “a biochemical change, functional impairment, or pathologic lesion that affects the performance of the whole organism, or reduces an organism’s ability to respond to an additional environmental challenge.” The multiple effects of acrolein on lung function indicate that there are a constellation of changes to lung function measurements consistent with the potential for adverse effects in humans. An alternate approach to our analysis would have been to model the end points as a group, because acrolein appears to lead to multiple effects on the lung. In this case, the approach we took in this analysis would lead to potentially underestimating the effects of acrolein exposure.

Consideration of individual susceptibility is also important. Individuals with existing respiratory impairment, such as chronic obstructive pulmonary disease or asthma, are more likely to exhibit adverse responses to irritants such as acrolein, and at potentially lower levels of exposure. Certain subpopulations (e.g., women, children, the elderly) may react differently following acrolein exposure, and our study uses data from healthy adult rats. The choice of a 10% rate of adverse effects in an unexposed population somewhat accounts for a susceptible portion of the population (e.g., 9% of children currently have asthma in the United States) [Federal Interagency Forum on Child and Family Statistics (FIFCFS) 2005] as well as the adjustments to the continuous exposure.

To estimate risks for continuous end points, it is necessary to define a response level considered adverse or abnormal. This can be challenging because continuous measurements of physiologic function capture both levels considered normal and those considered adverse. In some cases, any change above the background rate is considered adverse, as has been used in the U.S. EPA draft dioxin risk assessment (U.S. EPA 2004) for several outcomes such as thyroid function, immune or developmental effects, and enzyme (CYP1A1/1A2) induction. In other cases, a specific point estimate has been designated as a cutoff level for adverse responses: for example, obesity has been defined as a body mass index of ≥ 30.0 on the basis of studies that found a high risk for type 2 diabetes and cardiovascular disease in adults with a body mass index > 30 (National Institutes of Health 2000). Another method, the one used here, is to define adverse outcomes as at or above a specified percentile response of the baseline population. We chose the 90th percentile of controls as a reasonable definition of a baseline adverse response in the ratio of lung function parameters based on consideration of other health effects in humans (e.g., small for gestational age infants are defined as the 10th percentile of birth weight for gestational age), but more or less conservative choices for the cutoff do not substantially alter the estimated excess risks.

A separate analysis of combined estimated excess cancer risk for the identified carcinogenic hazardous air pollutants, using traditional cancer risk methods, found a median estimate of approximately two lifetime cases per 10,000 people exposed (Woodruff et al. 2000). Many of the assessments underlying the cancer risk estimates for hazardous air pollutants are based on a more robust literature (e.g., extensive occupational studies) than is available for acrolein. Comparing the amount of available data for these compounds suggests that further research on the health effects of acrolein would be useful to elucidate its potential risks.

Risk assessments for effects other than cancer generally rely on comparisons of estimated exposure levels to reference doses or reference concentrations. Although there are some exceptions for environmental contaminants with extensive epidemiologic data, noncancer risk assessments using laboratory animal data are almost exclusively based on the reference dose (RfD)/RfC model. Reliance on the RfD/RfC model has been based on the assumption that there exists a threshold of exposure for toxic chemicals, below which there is no appreciable risk (U.S. EPA 2002b). However, Clewell and Crump (2005) recently argued that the uncertainties involved at low doses for noncancer effects are no greater than those for the customary extrapolations used in cancer risk assessment, and that linear extrapolation of risks to low doses for noncancer effects is appropriate to inform regulatory decision-making (Clewell and Crump 2005).

There are several aspects of cancer and noncancer health effects associated with exposure to toxic chemicals that are similar and that support treating these health effects the same way from a risk-assessment perspective. The linear no-threshold model, frequently used in assessing excess cancer risk, is based on a number of biological assumptions. One is that the exposure to the carcinogen is adding to already existing biological processes (Clewell and Crump 2005). Given an appreciable background incidence of cancer in the human population and in control animals, there are other ongoing biological processes that independently contribute to the excess risk of cancer, and chemical exposures can enhance what is already occurring biologically. Another assumption is that exposure to an individual chemical is occurring in addition to other simultaneous or preexisting exposures to other chemicals (Crump et al. 1976). This other background exposure can contribute to the risk of cancer prior to exposure to the carcinogen of interest. If a carcinogen is thought to have a threshold in a scenario in which there are no other factors contributing to the development of cancer, the biological background and background exposures that occur in more realistic scenarios can effectively raise the starting point on the dose–response curve above any theoretical threshold. There is no reason to believe that exposures to chemicals for noncancer effects would act drastically differently. Several non-cancer diseases have appreciable background incidence in the population (e.g., cardiovascular effects, respiratory effects, reproductive effects), and the exposure to a particular chemical being assessed adds to existing exposures to other toxic chemicals. Therefore, exposures to chemicals with noncancer effects are likely to have the effect of shifting the response distribution, as shown in Figure 1.

In the particular example of the present acrolein analysis, respiratory conditions such as asthma are already at an appreciable level in the population. In addition, acrolein exposures occur simultaneously with exposure to other air pollutants, many of which are ubiquitous and have been demonstrated to cause respiratory effects (e.g., ozone and particulate matter). This analysis is consistent with the suggestion of Clewell and Crump (2005) that estimation of excess risks for noncancer effects could be conducted and presented in a manner similar to the analyses that have been conducted for potential carcinogens on a regular basis over the last two decades.

It is also important to consider how far the response is being extrapolated below the data. The greater the extrapolation, the greater its associated uncertainty. Mean and median national ambient acrolein exposures are 8.7 and 6.2 times lower, respectively, than the lowest HEC-converted dose used in this analysis. This is not a substantial extrapolation below the low end of the data, and we would not expect the shape of the curve to change drastically in this range. However, there is some increased uncertainty from estimating shifts at the tails of the distribution of response, which could be more uncertain than estimating shifts in the median of response. This is due to smaller numbers of responses at the ends of the distribution, and a change in response in a small number of animals at the ends of the distribution could have a relatively larger impact on the estimate of the 90th percentile in response.

A potential source of error in the risk estimate is the data transformation process. By transforming noncontinuous, 62-day exposures into continuous, yearly exposures, we assume that measured responses are independent of timing and intensity of exposure. The adjustments used in this analysis essentially assume that the response would occur at proportionally lower exposures if the exposures had been continuous over a year. Changing our assumptions would naturally alter our outcomes. Two examples of this are *a*) adjusting the 62-day exposure time to a 90-day period (the usual subchronic period) instead of a 365-day period; or *b*) adjusting to a 90-day subchronic period and also applying a 10-fold uncertainty factor to account for potential differences between subchronic and chronic exposures. If we had adjusted the exposure duration to standard subchronic length, the slope of the dose–response relationship between predicted adverse responses and exposures would be about 4 times lower (i.e., less steep). This would reduce the amount of predicted risk. However, if we had adjusted exposures to sub-chronic durations and then applied a 10-fold uncertainty factor to account for adjusting from subchronic to chronic exposures, this slope would be about 2.5 times higher than we estimated (i.e., a steeper dose response); thus, using an uncertainty factor on a subchronic exposure measure would increase the predicted risk. The transformation method we selected is therefore well within a plausible range.

The excess risks presented here are based on estimated ambient outdoor exposures; however, indoor acrolein levels can be 2–20 times higher than outdoor concentrations, especially in areas with high levels of environmental tobacco smoke (U.S. EPA 2003b). Because most individuals spend the majority of their time indoors (Klepeis et al. 2001; Weisel 2002), excess risks from acrolein exposure could well be higher than those predicted here.

The present analysis provides an example of how a noncancer risk assessment could be undertaken using a dose–response, nonthreshold model, similar to standard approaches for cancer risk assessment. This type of analysis is not likely to involve more uncertainty than recommended methods for cancer risk assessment, and it has the potential to provide more detailed information than traditional noncancer risk assessments. Using this method, we found that ambient acrolein levels may be contributing to decreased respiratory function. These results suggest increased use of nonthreshold models for noncancer risk assessment should be explored further.

## Figures and Tables

**Table 1:** Summary of lung function data (mean ± SD) from Costa et al. (1986).

	Acrolein concentration in air (ppm)				
Measure	All (*n* = 77)	0.0 (*n* = 24)	0.4 (*n* = 23)	1.4 (*n* = 21)	4.0 (*n* = 9)
RV (cm^3^)	1.67 (1.02)	1.34 (0.57)	1.46 (0.86)	1.51 (0.53)	3.48 (1.39)
TLC (cm^3^)	10.74 (1.93)	10.13 (1.02)	9.97 (1.05)	10.57 (1.24)	14.71 (2.17)
RV/TLC	0.15 (0.06)	0.13 (0.05)	0.14 (0.07)	0.14 (0.04)	0.23 (0.06)
C_dyn_ (cm H_2_O^−1^)	0.23 (0.08)	0.24 (0.07)	0.24 (0.10)	0.20 (0.06)	0.26 (0.10)
FRC (cm^3^)	3.55 (1.43)	3.06 (0.46)	3.06 (0.40)	3.26 (0.56)	6.81 (1.97)
sC_L_ (cm^3^/cm H_2_O)	0.07 (0.03)	0.08 (0.03)	0.08 (0.03)	0.06 (0.02)	0.04 (0.02)

Abbreviations: C_dyn_, dynamic compliance; FRC, functional reserve capacity.

**Table 2:** Estimated acrolein concentrations (μg/m^3^) across the United States in 1999.^a^

Percentile	National average	Urban counties	Rural counties
5	0.0087	0.017	0.0034
10	0.015	0.027^b^	0.0054
25	0.034^b^	0.052^b^	0.011
50	0.077^b^	0.094^b^	0.021^b^
75	0.14^b^	0.16^b^	0.035^b^
90	0.26^c^	0.29^c^	0.061^b^
95	0.41^c^	0.44^c^	0.091^b^

a Data from U.S. EPA’s 1999 *National-Scale Air Toxics Assessment* (U.S. EPA 2006).

b Value > RfC.

c Value > 10 times higher than the RfC for acrolein (0.02 μg/m^3^).

**Table 3:** Percent change in lung function parameters above background for the range of modeled acrolein concentrations across the United States, 1999.

		Percent change^a^	
Acrolein concentration (μg/m^3^)	Percentile^b^	sC_L_	RV/TLC
0.0087	5	−0.060	1.4 × 10^−6^
0.034	25	−0.23	5.0 × 10^−5^
0.077	50	−0.53	4.3 × 10^−4^
0.14	75	−0.96	2.1 × 10^−3^
0.41	95	−2.8	3.4 × 10^−2^
RfC^c^		−0.14	1.25 × 10^−5^

a Percent change in lung function parameter is defined as the [parameter (exposed) – parameter (unexposed) (baseline)] ÷ [parameter (baseline)] × 100; parameters are estimated from the regression equations presented in Table S1 (Supplemental Material available online at http://www.ehponline.org/docs/2006/9467/suppl.pdf).

b Percentile of estimated acrolein concentration distribution across all census tracts.

c The current RfC for acrolein is 0.02 μg/m^3^.
